# Herbal medicine (Danggui Liuhuang decoction) for managing menopausal symptoms: A protocol of systematic review of randomized clinical trials

**DOI:** 10.1097/MD.0000000000009735

**Published:** 2018-01-26

**Authors:** Ji Hee Jun, Hye Won Lee, Junhua Zhang, Fengwen Yang, Myeong Soo Lee

**Affiliations:** a Clinical Research Division; b KM Convergence Research Division, Korea Institute of Oriental Medicine, Daejeon, Republic of Korea; c Evidence Based Medicine Center, Tianjin University of Traditional Chinese Medicine, Tianjin, China; d Department of Preventive Medicine, College of Korean Medicine, Daejeon University, Daejeon, Republic of Korea.

**Keywords:** Danggui Liuhuang decoction, herbal medicine, menopausal symptoms

## Abstract

**Background:**

Danggui Liuhuang (DLH) decoction is a traditional herbal medicine that is widely used in East Asia to treat menopausal symptoms. Most of the available clinical trials that investigated DLH decoction have been included in this review. The objectives of this protocol are to provide the information of how to evaluate the effectiveness and safety of DLH decoction for the treatment of menopausal symptoms.

**Methods and analysis:**

Fourteen databases will be searched from inception until February 2018. We will include randomized controlled trials (RCTs) testing any type of DLH decoction. All RCTs investigating DLH decoction or modified DLH decoction will be included. The methodological quality of the RCTs will be evaluated using the Cochrane's risk of bias assessment tool.

**Ethics and dissemination:**

The full systematic review will be published in a peer-reviewed journal. The review will also be disseminated electronically and in print. Updates of the review will be conducted to inform and guide healthcare practice and policy.

**Trial registration number:**

PROSPERO 2017 CRD42017079189

## 1 Introduction

Menopausal symptoms are a result of a decrease in the secretion of female sex hormones and gradual decline in ovarian function. Two main types of symptoms are associated with menopause, namely, consistently appearing symptoms and less consistently appearing symptoms. Consistent symptoms include vasomotor indications (hot flashes, diaphoresis) and vaginal dryness; less consistent symptoms include sleep disturbances, mood changes, urinary tract symptoms, sexual problems, and other bodily indications.^[1]^

Hormone replacement therapy (HRT) is widely used for treating menopausal symptoms.^[2–4]^ However, the prolonged use of HRT increases the risk of adverse effects (AEs). HRT can cause stroke, dementia, and mild cognitive impairments.^[5]^ Additionally, longer durations of HRT are associated with a higher risk of hearing loss.^[6]^ Recently, many researchers are studying alternative methods that can be used to replace HRT.^[7,8]^

Herbal medicine is widely used in East Asian countries such as Korea, China, and Japan. Additionally, the use of herbal medicine is expected to increase, as women suffering from menopausal symptoms are seeking alternative therapies due to concerns regarding AEs associated with HRT.^[7,9]^ However, scientific evidence for the effectiveness and safety of any medicine is needed. The compound known as Danggui Liuhuang (DLH) decoction in Korea and China has been prescribed for the treatment for night sweats, fever, red face, distress, dry mouth, and constipation. DLH decoction consists of 7 medicinal herbs, namely, *Angelicae Gigantis Radix, Astragali Radix, Coptidis Rhizoma, Rehmanniae Radix Crudus, Rehmanniae Radix Preparata, Phellodendri Cortex,* and *Scutellariae Radix.*^[10,11]^ DLH decoction has been reported to have immunomodulatory^[12]^ and anti-inflammatory effects.^[13]^

Currently, except for reviews on various types of herbal medicine,^[14–17]^ no systematic review is available for DLH decoction. The objective of this systematic review was to summarize and critically assess evidence from randomized controlled trials (RCTs) that have investigated the use of DLH decoction for the treatment of menopausal symptoms.

## 2 Methods

### 2.1 Study registration

This protocol review has been registered on PROSPERO 2017 CRD42017079189.

### 2.2 Data source

Fourteen databases will be searched from their inception to February 2018, including PubMed, EMBASE, the Cochrane Central Register of Controlled Trials (CENTRAL), AMED, and CINAHL. We will also search 6 Korean medical databases (OASIS, Korean Traditional Knowledge Portal, Korean Studies Information Service System, Korea Med, Korean Medical Database, and DBPia) and 3 Chinese databases, including CNKI, Wanfang, and VIP. We will use the following search terms: (Danggui Liuhuang decoction OR Danggui Liuhuang tang OR Danggui Liuhuang) AND (climacteric OR menopause OR menopausal OR perimenopause OR peri-menopausal OR perimenopause period OR menopausal syndrome OR climacteric syndrome OR female climacteric syndrome). Searches will be conducted in Korean, English, and Chinese.

### 2.3 Study selection

#### 2.3.1 Types of studies

We will include all RCTs and quasi-RCTs comparing any type of DLH decoction with herbal medicine or Western medicine. Case studies, qualitative studies, uncontrolled trials, and reviews will be excluded, as will be trials that failed to provide detailed results.

### 2.4 Types of participants

Participants will include perimenopausal or postmenopausal women experiencing menopausal symptoms. We will exclude women in menopause secondary to surgery or chemotherapy and/or radiotherapy who were experiencing climacteric symptoms.

#### 2.4.1 Types of interventions

Studies using all types of DLH decoction or modified DLH decoction will be included, as will be studies comparing the effects of DLH decoction alone or in combination with Western medicine. DLH decoction includes the following 7 ingredients: *Angelicae Gigantis Radix, Astragali Radix, Coptidis Rhizoma, Rehmanniae Radix Crudus, Rehmanniae Radix Preparata, Phellodendri Cortex,* and *Scutellariae Radix*. Modified DLH decoctions are defined as a single decoction with one or more supplemental herbs. We will include various types of medications, such as tablets, capsules, pills, powders, and extracts. We will exclude DLH decoction combined with other types of therapies, such as acupuncture, moxa, and cupping. The control groups will be included Western medicine, placebo, or no treatment.

#### 2.4.2 Types of outcome measurements

The primary outcomes will be response rate of symptom reduction and menopause rating scale (the Kupperman index). The secondary outcomes will be AEs.

### 2.5 Data extraction and risk of bias assessment

#### 2.5.1 Data extraction

All trials from the electronic database searches will be reviewed by 2 authors (JHJ and HYL) who will select the relevant trials through a review of the titles and abstracts. They will extract data according to predefined criteria. Information such as the participants, interventions, outcomes, and results were obtained from each report. We will use GRADEpro GDT software (https://gradepro.org/) to create a Summary of Findings table. Any disagreements will be resolved by discussing with the 2 authors (JHH and HYL) and an arbiter (MSL). The authors of the included trials will be contacted for clarification if necessary. Data will be collected from the included trials by 2 authors (JHJ and HYL).

#### 2.5.2 Risk of bias

The risk of bias will be assessed using the “risk of bias” assessment tool from the Cochrane Handbook (version 5.3.), which examines 7 domains of study quality, namely, random sequence generation, allocation concealment, blinding of participants and personnel, blinding of outcome assessment, incomplete outcome data, selective reporting, and other bias.^[18]^ We will use “L,” “H,” and “U” as a key for assessing the risk of bias, with “L” indicating a low risk of bias, “H” indicating a high risk of bias, and “U” indicating that the risk of bias is unclear. MSL will make the final decision as an arbiter for any unresolved disagreements.

### 2.6 Data analysis

All of the statistical analyses will be performed using Review Manager Software (Version 5.3). For dichotomous data, we will present the treatment effects as relative risk (RR) with 95% confidence intervals (CIs). For continuous data, we will use the mean difference (MD) with 95% confidence intervals to present the treatment effects. We will convert other forms of data into MDs.

#### 2.6.1 Assessment of heterogeneity

If a meta-analysis is possible, we will use the *I*^2^ statistic for quantifying inconsistencies across the included studies. A result 50% cut-off point would represent substantial heterogeneity. If heterogeneity is observed, we will conduct subgroup analyses.^[18]^

#### 2.6.2 Subgroup analysis and the investigation of heterogeneity

If studies and data are sufficient, subgroup analyses will be conducted according to:

Type of control intervention (e.g., Western medicine, placebo, no treatment or usual care)

#### 2.6.3 Sensitivity analysis

We will use sensitivity analyses to investigate suspected funnel plot asymmetry. Sensitivity analysis will be conducted according to the following criteria:

1. Methodological qualities (sequence generation, allocation concealment, or blinding in the assessment of outcomes and symptom severity).
2. Sample size (more or less than 40 participants in each group).

In the analysis, we will exclude high risk of bias studies and compare the results with those using the worst-case scenario to combine studies. Then we will have a discussion to decide whether the high risk of bias studies should be excluded on the bias of sample size, strength of evidence and influence on pooled effect size.

#### 2.6.4 Assessment of reporting biases

If more than 10 studies are available, we will conduct funnel plot for publication bias and small study effects using Egger's method. Funnel plot asymmetry is certainly not same as publication bias. We will attempt to distinguish the possible reasons for the asymmetry, therefore, included poor methodological quality and true heterogeneity of studies.^[19]^

## 3 Discussion

There have been no systematic reviews of DLH decoction for menopausal symptoms so far. The fully completed systematic review will provide a summary of the current state of evidence regarding the effectiveness of the DLH decoction in managing the menopausal symptoms. The review will be useful to patients and healthcare providers in the field of women's health as well as patients.
